# Optical Properties of Bismuth Tellurite Based Glass

**DOI:** 10.3390/ijms13044623

**Published:** 2012-04-11

**Authors:** Hooi Ming Oo, Halimah Mohamed-Kamari, Wan Mohd Daud Wan-Yusoff

**Affiliations:** Physics Department, Faculty of Science, University Putra Malaysia, 43400 UPM Serdang, Selangor, Malaysia; E-Mails: hooimingoo@gmail.com (H.M.O.); wmdaud@science.upm.edu.my (W.M.D.W.-Y.)

**Keywords:** tellurite glass, optical band gap, optical properties

## Abstract

A series of binary tellurite based glasses (Bi_2_O_3_)*_x_* (TeO_2_)_100−_*_x_* was prepared by melt quenching method. The density, molar volume and refractive index increase when bismuth ions Bi^3+^ increase, this is due to the increased polarization of the ions Bi^3+^ and the enhanced formation of non-bridging oxygen (NBO). The Fourier transform infrared spectroscopy (FTIR) results show the bonding of the glass sample and the optical band gap, *E*_opt_ decreases while the refractive index increases when the ion Bi^3+^ content increases.

## 1. Introduction

Tellurite glass is a good material used in lasers and nonlinear applications, photonic applications and communication applications [1]. Tellurite glass is often used because it is stable at room temperature, has good thermal, optical and electric properties [2,3]. This glass has a low photon energy, a high linear and non-linear refractive index, and it also can be used in photorefractive materials, nonlinear devices, up-conversion lasers and optical amplifiers [4–6]. Metal oxides such as Bi_2_O_3_, Sb_2_O_3_, MoO_3_ and Nb_2_O_5_ have been added to the tellurite glass system in order to enhance the optical behavior [7,8]. Many studies on tertiary tellurite based glass have been done by various authors [9–16]. For example, Tiefeng *et al*. (2011) [14] studied the optical non linear properties of TeO_2_–Bi_2_O_3_–BaO glass, and was found that an increase of the ion bismuth content and a decrease of ion barium content increased the non linear properties of the glass as bismuth dissolved in the tellurite glass matrix.

Bismuth (iii) oxide is used in optical fiber and ceramic materials because it can provide a high refractive index and low temperatures [17]. In addition, it is also used in the electronic field due to it having a high valence cation of low field strength and high polarizability [18]. In this paper, the optical and physical properties were determined with different ion bismuth contents of tellurite base glass. Optical properties such as the optical band gap and refractive index of binary bismuth tellurite glass were studied. In addition, the density and molar volume of a tellurite glass system was determined.

## 2. Results and Discussion

### 2.1. XRD

**Figure 1:**
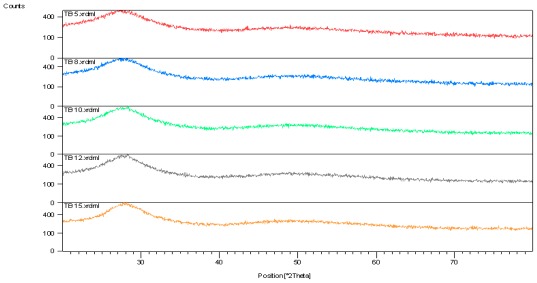
X-ray diffraction (XRD) analysis of the (Bi_2_O_3_)*_x_*(TeO_2_)_100−_*_x_* glass system.

The XRD results (Figure 1) show that the bismuth tellurite glass is in an amorphous phase. Sample TB 5 (refer to Table 1) is a transparent glass of a dark orange color, while TB 8, TB10, TB12 and TB15 are transparent glasses of a yellow color.

### 2.2. Density and Molar Volume

**Figure 2:**
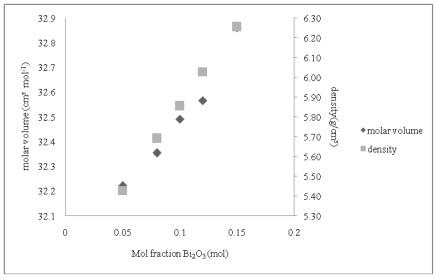
Density and molar volume with mole fraction of bismuth (iii) oxide of the (Bi_2_O_3_)*_x_*(TeO_2_)_100−_*_x_* glass system.

The density of the glass samples increases as the bismuth content increases. The densities of the bismuth tellurite glasses range from 5.43 g/cm^3^ to 6.26 g/cm^3^, as shown in Table 1. Figure 2 shows the molar volume and the density increase proportional to the bismuth content. The molar mass of the bismuth (iii) oxide (465.96 g/mol) is heavier than the molar mass of tellurite oxide (159.60 g/mol) and hence, the glass matrix becomes more dense when Bi^3+^ ions are added into the glass network [19]. In addition, the increase in molar volume is due to the atomic radius of Bi^3+^ (1.70 Å ) which is higher than that of tellurite (1.60 Å ). Usually, the density of the glass changes in the inverse direction of the molar volume, but in this study, the density and molar volume increase with the bismuth contents, this anomaly was also found by Saddek *et al*. (2007) and Halimah *et al*. (2010) [20,21].

### 2.3. FTIR Results

**Figure 3:**
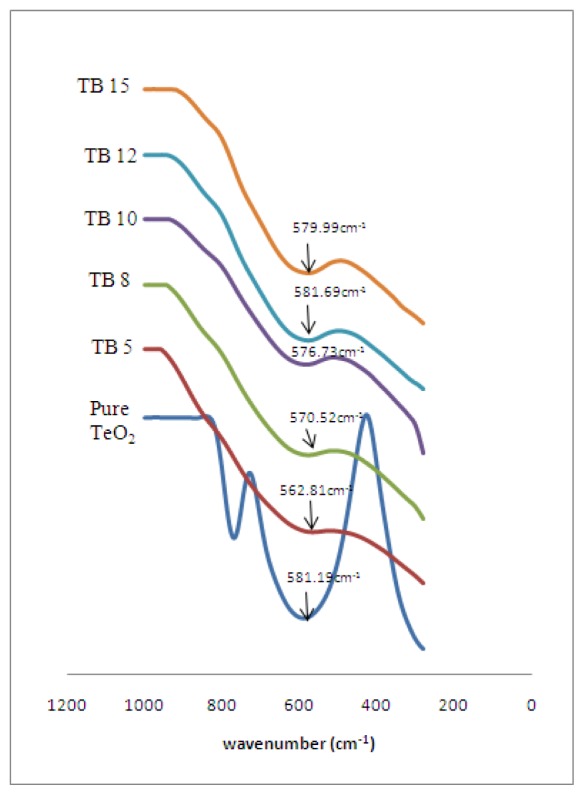
FTIR analysis of (Bi_2_O_3_)*_x_*(TeO_2_)_100−_*_x_* glass system.

As shown in Figure 3, the FTIR analysis shows that the TeO_2_ is the framework former while Bi_2_O_3_ is the glass modifier in the bismuth tellurite glasses system. Table 2 shows the ranges of the wave numbers for difference mode. The wave number for the bismuth tellurite glass system is slightly shifted to the lower wave numbers when the ion Bi^3+^ concentration is increased. There is a diffuse band in the range of 500–600 cm^−1^, due to the disordered structure; this is considered as the vibration modes of both the TeO_3_ and the TeO_4_ entities [22]. The vibration modes of the bonds for TeO_3+1_ polyhedra is also found at bands around 580 cm^−1^ where the TeO_3+1_ polyhedra consists of an intermediate coordination of the tellurium atoms between 3 and 4 [17], this is found in the all glass samples. On the other hand, the shoulder form in the range of 800–900 cm^−1^ in TB8, TB10, TB12 and TB15 is due to the symmetrical stretching vibration of the Bi–O bonds in BiO_3_ units. This band is not observed in the TB5 because the Bi^3+^ ions present in this glass sample contain non-bridging oxygen in the form of [BiO]^−^ defects. As the ion Bi^3+^ content increases, bismuth oxide as the network modifier ion modifies the glass structure and enhances the breaking of axial Te–O–Te linkages in the trigonal bipyramids [TeO_4_] (tbp) and creates the formation of trigonal pyramid [TeO_3_] (tp) units and non-bridging oxygen.

### 2.4. Refractive Index and Optical Absorption

When the bismuth content increases, the refractive index measured at the wavelength of 632.8 nm increases from 1.97 to 2.12. The refractive index increase is due to the ion Bi^3+^ which has a high polarity that can break the bridging oxygen [BO] to non-bridging oxygen [NBO]. In addition, non-bridging oxygen also has an effect on the refractive index, because of the polarity of non-bridging oxygen (NBO) is higher than that of bridging oxygen [29]. As a result, Bi_2_O_3_ will affect the TeO_2_ based glasses and forms highly polarized non-bridging oxygen ions at the terminals of Te–O bonds.

The optical absorption spectra were taken in the ranges of 350 to 500 nm. For the optical band gap in this bismuth tellurite glass system, the optical band gap energy, *E*_opt_ decreases when the ion bismuth content increases as shown in Table 3.

**Figure 4:**
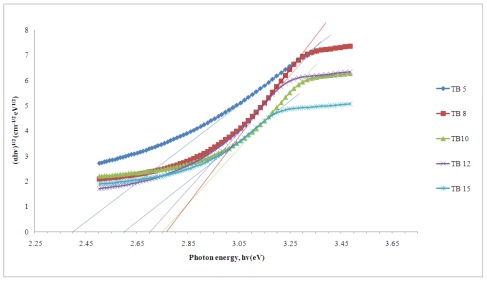
Plot of (αhv)^1/2^ *versus hv* for indirect band gap of (Bi_2_O_3_)*_x_*(TeO_2_)_100_**_−_***_x_* glass system.

The optical band gap energy is determined by using the following equation [30]

(1) $$\alpha hv=A{\left(hv-E_{\text{opt}}\right)}^{n}$$

where *α* is the absorption coefficient, *hv* is the incident photon energy, *A* is a constant and *E*_opt_ is the optical band gap. Values of *n* are 2 and 1/2 for direct and indirect transitions, respectively. The indirect band gap as a function of photon energy for (Bi_2_O_3_)*_x_*(TeO_2_)_100−_*_x_* glass system is plotted and shown in Figure 4.

**Figure 5:**
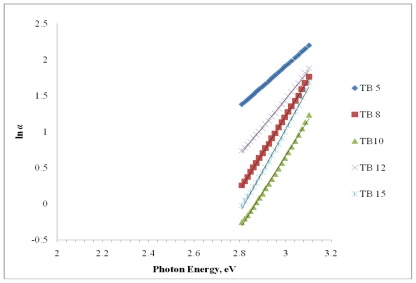
Plot of ln (*α*) *versus hv* for Urbach energy of (Bi_2_O_3_)*_x_*(TeO_2_)_100_**_−_***_x_* glass system.

According to Sayed (2005), amorphous or glassy materials consist of a band tailing in the forbidden energy band gap. The band tailing might arise from random fluctuations of the internal disorder in the amorphous materials. So it can be estimated using the Urbach equation shown as follows [31]

(2) $$\alpha (v)=B\;exp\;(hv/\Delta E_{g})$$

where *B* is a constant and Δ*E* is the width of the band tail of the electron states. From Figure 5, Urbach energy, *E*_g_ is determined from the slope of plot ln (*α*) *versus hv*.

The range of the indirect optical band gap decreases from 2.40 eV to 2.60 eV and 3.00 eV to 3.02 eV for the direct band gap as the concentration of ion Bi^3+^ increases in the bismuth tellurite glass system. However, sample TB5 has a low bismuth content and a low energy band gap because of the formation of TeO_2_ linkages in Te–O where bridging oxygen is more frequent than non-bridging oxygen. Shifting of the absorption band to a lower energy can be related to the formation of non-bridging oxygen (NBO) which binds exited electrons of non-bridging oxygen less tightly than bridging oxygen [10], and this cause the glass network to become less rigid (Rajendan, 2003) [27]. Another possibility is due to the high polarizability of the Bi^3+^ ions which can be attributed to the empty d orbitals of the corresponding cations and also their high coordination number towards oxide ions. The coordination number of TeO_2_ changes from 4 to 3 while the coordination number of Bi_2_O_3_ changes from 3 to 6, which then forms a BiO_6_ octaherdal structural unit with the addition of Bi_2_O_3_ into the glass system. According to Yanfei (2008) [16], less tight oxygen anions were allowed when the amount of non-bridging oxygen increased, This was because of an increase in Lewis basicity of oxide ions, which form stronger covalent Te–O bonds in TeO_3_ units, and so allow less tight oxygen anions in the glass network. Hence the more the Bi_2_O_3_ content increases, the fewer tightly bound oxygen anions (valence electrons) are found and the greater the decrease of the optical band gap energies.

**Figure 6:**
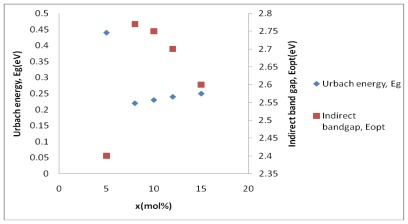
The optical band gap energy, *E*_opt_ and Urbach energy, *E*_g_ of (Bi_2_O_3_)*_x_*(TeO_2_)_100−_*_x_* glass system.

The optical band gap energy is opposite to the Urbach energy, which can be attributed to the formation of non-bridging oxygen (Figure 6). The optical band gap energy and Urbach energy values for glass systems are found to lie between 3.10–3.00 eV and 0.25–0.44 eV, respectively. Rajendran (2003) [27] reported that the addition of Bi_2_O_3_ glass modifier would make the glass network more loosely packed (breaking the O–Te–O chains to form TeO_3_ units and non-bridging oxygen) and therefore produce unstable glass. When the concentration of ion Bi^3+^ is increased, the appearance of TeO_3_ units and non-bridging oxygen in the glass networks increases. As the concentration of the ion modifier Bi^3+^ increases, non-bridging oxygen and TeO_3_ units in the glass structure increase and as a result, the Urbach energy for this glass system also increases.

## 3. Experimental

The raw materials of tellurium dioxide, TeO_2_ (Aldrich 99.5%), and bismuth (iii) oxide Bi_2_O_3_ (Aldrich 99.0%) were used to synthesize the glass sample. The glass sample was prepared by using a melt quenching method [32,33]. The bismuth (iii) oxide, *x* = 5, 8, 10, 12, 15 in mol% was added into the tellurite oxide and was weighed using a digital weighing machine with an accuracy of ±0.01 g and then mixed together by using a mortar and pestle. The mixture was poured into an alumina crucible and put into an electric furnace set at 100 °C for a period of 30 min. The alumina crucible was used because it can withstand high temperatures and does not react with the raw materials during the melting process as opposed to porcelain crucibles. The temperature was then increased by 10 °C/min until 900 °C and the then maintained for 2 h. When the melting process was completed, the molten liquid was cast into a stainless steel cylindrical shape mould which had been preheated at 400 °C for 30 min. The sample was annealed at 400 °C in a second furnace for 1 h, then the furnace was turned off. The glass sample was cut at a thickness of about 2 mm for the required measurements [33].

The density of the glass samples was measured using the Archimedes principle, and distilled water was used as the immersion liquid. The optical properties such as refractive indices (*n*) were determined by using EL X-02C high precision ellipsometer. The structure of the bismuth tellurite glass was investigated using X-ray diffraction (XRD) and Fourier transform infrared spectroscopy (FTIR). The optical absorption of the bismuth tellurite glass was measured using UV visible spectroscopy and powder BaSO_4_ was used as the reference for the UV visible spectroscopy. The wavelength was set from 2600 nm to 220 nm, and the internal spacing was 0.2 nm, using a medium scan speed for glass samples.

## 4. Conclusions

The density and molar volume of the glass sample increase due to the fact that the atomic mass of bismuth ions is higher than that of tellurite ions, and that the atomic radius of bismuth is also greater than that of tellurite ions. Additionally, the refractive index increases due to the increase of polarity of the Bi^3+^ ion content in tellurite based glasses. In addition, based on the FTIR results, the TeO_2_ is the glass former while Bi_2_O_3_ is the glass modifier in bismuth tellurite glass systems, and the wave number shifts to a low frequency when the bismuth content increases. The optical band gap shifts to a low energy while the Urbach energy shifts to high energy when non-bridging oxygen (NBO) increases as bismuth content increases.

## Figures and Tables

**Table 1:** Molar mass, density and molar volume of the (Bi_2_O_3_)_x_(TeO_2_)_100−x_ glass system.

Samples	*x* (mol%)	Molar mass (g/mol)	Density (g/cm^3^)	Molar Volume (cm^3^/mol)
TB5	5	174.92	5.43	32.22
TB8	8	184.11	5.69	32.35
TB10	10	190.23	5.85	32.49
TB12	12	196.36	6.03	32.56
TB15	15	205.55	6.26	32.86

**Table 2:** The ranges of the wave numbers for difference mode.

Wavenumber(cm^−1^)	Mode
400–600	Bi–O–Bi + Bi–O in BiO_6_ octahedral [23]
650–660	Te–Oax in [TeO_4_] [24,25]
775	Te–Oeq in [TeO_4_] [26,27]
633, 695	(shoulders) Te-O^−^ [10]
600–610	Bi–O^−^ stretch in BiO_6_ units [28]
860–865	Bi–O vibration in distorted BiO_6_ units [11]

**Table 3:** Direct optical bandgap, indirect optical bandgap and Urbach energy of (Bi_2_O_3_)_x_(TeO_2_)_100−x_ glass system.

Sample glass	Indirect bandgap, *E*_opt_ (eV)	Direct bandgap (eV)	Urbach energy, *E*_g_ (eV)	Refractive index in wavelength 632.8 nm, *n*
TB 5	2.40	3.02	0.44	1.97
TB 8	2.77	3.10	0.22	1.99
TB 10	2.75	3.08	0.23	2.02
TB 12	2.70	3.04	0.24	2.07
TB 15	2.60	3.00	0.25	2.12
